# Maternal multiple micronutrient supplementation in rural Pakistan increased some milk micronutrient concentrations, but not infant growth, at three-months postpartum: a randomized controlled trial substudy

**DOI:** 10.1016/j.ajcnut.2025.05.019

**Published:** 2025-05-21

**Authors:** Jo-Anna B Baxter, Yaqub Wasan, Allison I Daniel, Kehkashan Begum, Amjad Hussain, Junaid Iqbal, Susanne Aufreiter, Megan R Beggs, Lauren Duan, Adrianna Greco, Carolina Huang, Sajid Soofi, Robert HJ Bandsma, Zulfiqar A Bhutta, Deborah L O’Connor

**Affiliations:** 1 Department of Nutritional Sciences, University of Toronto, Ontario, Canada; 2 Centre for Global Child Health, Hospital for Sick Children, Ontario, Canada; 3 Centre of Excellence in Women and Child Health, Aga Khan University, Sindh, Pakistan; 4 Translational Medicine Program, Hospital for Sick Children, Ontario, Canada; 5 Division of Gastroenterology, Hepatology and Nutrition, Hospital for Sick Children, Ontario, Canada; 6 Institute for Global Health and Development, Aga Khan University, Sindh, Pakistan; 7 Department of Paediatrics, Mount Sinai Hospital, Ontario, Canada

**Keywords:** breastmilk micronutrient concentrations, infant growth, multiple micronutrient supplementation, breastfeeding, maternal nutrition, pregnancy

## Abstract

**Background:**

In Pakistan, maternal micronutrient deficiencies are highly prevalent, and stunting affects 43% of infants by 6-mo postpartum. Human milk composition for some micronutrients can be negatively affected by suboptimal maternal nutrition; however, it is unknown whether this affects infant growth.

**Objectives:**

We aimed to determine whether mothers receiving multiple micronutrient supplements (MMSs) compared with standard of care had *1)* greater concentrations of iodine, vitamins A, E, and B12, and folate in their milk at 3-mo postpartum; and *2)* improved growth of their offspring. Associations between milk micronutrients and infant growth were also explored.

**Methods:**

This substudy was nested within a district-based, cluster-randomized, controlled trial (MaPPS Trial; 25,477 females) with the primary aim of evaluating whether maternal MMS (preconception: twice-weekly, pregnancy and postpartum: daily, to 6-mo postpartum) compared with the standard of care (preconception: no intervention; pregnancy and postpartum: daily iron and folic acid supplementation, to 6-mo postpartum) in rural Pakistan improved infant birthweight. Substudy mother-infant dyads (*n* = 186) were recruited if infants were term-born and predominantly or exclusively breastfed. Milk micronutrient concentrations were compared to reference values derived from mother’s milk [mother’s milk adequacy estimates (MAEs)].

**Results:**

MMS increased milk iodine and vitamin A concentrations, but not vitamins B12 or E, nor folate. Importantly, few milk sample micronutrients in either arm were above existing MAEs. MMS compared to standard of care did not improve infant growth. Independent of allocation, having all 5 milk micronutrients below MAEs was associated with decreased infant length-for-age z-score (β: –0.39, 95% CI: –0.73, –0.04; *P* = 0.03).

**Conclusions:**

In a population with maternal micronutrient deficiencies, providing maternal MMS was not associated with milk micronutrient concentrations above MAEs; however, infants born to mothers with milk below MAEs for all investigated micronutrients appeared to experience poorer growth. Further research is needed to understand longer-term implications, if any.

**Clinical Trial Registry number and website:**

ClinlicalTrials.gov: NCT04451395 (https://clinicaltrials.gov/study/NCT04451395) and NCT03287882 (https://clinicaltrials.gov/study/NCT03287882).

## Introduction

Although child survival has increased worldwide, child malnutrition remains a major concern in resource-limited countries. Nearly half of children risk not reaching their developmental potential in such settings, largely due to poor nutrition and its sequelae [1]. Notably, early wasting and linear growth retardation remain an intractable and stagnant global issue [2,3].

Exclusively breastfeeding for the first 6-mo postpartum is considered optimal to support infant growth and development [4,5]. Human milk is uniquely suited to nourish infants and establish a healthy life trajectory, as it provides readily bioavailable and easily digestible nutrients, as well as bioactive compounds known to reduce risk of infection [3,6]. However, milk micronutrient composition can vary with maternal micronutrient status, particularly if diet quality is limited and widespread micronutrient deficiencies exist [7,8]. Because the first months of early postnatal life are a period of rapid development of the immune system, lean body mass accretion, and brain development, low intakes of micronutrients that are essential for these processes, such as iodine, vitamin A, vitamin E, folate, and vitamin B12, have the potential to critically impair growth and cognitive and motor development—with long-term consequences [[8], [9], [10], [11], [12]].

In Pakistan, the prevalence of childhood stunting is high [i.e., length-for-age z-score (LAZ) <−2 SD], with 43% of infants not achieving the associated cut-off by 6-mo postpartum [13]. Lactation is widely practiced, with 94% of infants under 2 y receiving human milk; however, only 54% appear to be exclusively breastfed for the first 6-mo postpartum [14]. National data show that reproductive-age females can experience multiple micronutrient deficiencies, including iodine (urinary iodine 20–49 μg/L and <20 μg/L: 13.2% and 4.7%, respectively, indicative of moderate and severe deficiency), vitamin A (serum retinol <0.70 μmol/L: 27%), folate (serum folate <10 nmol/L: 51%), and vitamin B12 (serum cobalamin <150 pmol/L: 20%) [13,15]. We previously reported that dietary quality is very limited among young females in rural Pakistan, with only 14% consuming a diet likely to meet their micronutrient needs (≥5 food groups/d) [16], and 91% experiencing ≥1 micronutrient deficiency [17]. How these factors relate to mature human milk composition and infant growth in this setting, and the potential beneficial effect of providing multiple micronutrient supplementation (MMS) during both pregnancy and lactation, has not been studied.

Leveraging an ongoing district-wide, randomized trial in a population with highly prevalent maternal micronutrient deficiencies, this study aimed to investigate the effect of maternal MMS into postpartum compared to the standard of care on *1)* milk micronutrient composition and *2)* infant growth at 3-mo postpartum. Associations between milk micronutrient composition and infant growth were also explored.

## Methods

### Study design and participants

This substudy (ClinicalTrials.gov Identifier: NCT04451395) was nested within a district-based, 2-arm, prospective, cluster-randomized, controlled trial of MMS compared with the standard of care, the Matiari emPowerment and Preconception Supplementation (MaPPS) Trial (ClinicalTrials.gov Identifier: NCT03287882). The primary aim of the MaPPS Trial was to assess the effect of MMS during preconception and pregnancy on the prevalence of low birthweight (<2500 g). The MaPPS Trial commenced in July 2017, and primary outcome data collection completed March 2021. In total, 25,447 females were enrolled in the MaPPS Trial, and 4,163 live births were observed. Mother-infant dyads were followed until 12-mo postpartum, with infant growth measured at birth and 1, 3, 6, 9, and 12 mo postpartum. The substudy reported in this article was conducted from August 2020 to April 2021.

The design and methods for the MaPPS Trial have been published [18]. Briefly, adolescent and young females (aged 15–23 y) living in Matiari District were randomly assigned to receive in the experimental arm of the trial an MMS, provided as a tablet [United Nations International Multiple Micronutrient Preparation (UNIMMAP) formulation [19]] from preconception (twice-weekly), and continued through pregnancy (daily) to 6-mo postpartum (daily). UNIMMAP MMS was added to the WHO Essential Medicines List in 2021, and recognized as an MMS formulation to meet the specific micronutrient requirements of pregnancy in low- and middle-income countries (LMICs) [20,21]. The control arm of the trial reflected the current standard of care provided through the existing public health system in Pakistan, per traditional WHO antenatal care recommendations [22], which included daily iron and folic acid supplementation (60 mg elemental iron and 400 μg of folic acid daily) during pregnancy. Detailed description of the supplement frequency, duration of use, and formulation is presented in Supplemental Tables 1 and 2. Allocation was assigned by an independent statistician using a computer-generated stratification sequence. Outcome assessors were blinded to study allocation.

### Eligibility

At the routine MaPPS Trial 3-mo visit, mothers were asked if they would be interested in being screened for participation in the milk substudy. Eligibility criteria for the substudy were as follows: *1)* infant term-born (>37 wk of gestation); *2)* infant 3 mo ± 30 d old; *3)* maternal willingness to provide a complete breast expression; and *4)* exclusive or predominant breastfeeding. Items 1 and 2 were determined from MaPPS Trial data records and confirmed in-person. The WHO definition of “predominant breastfeeding” was used, meaning that the infant's predominant source of nourishment was human milk, although the infant may have also received liquids (e.g., water, water-based drinks, fruit juice), ritual fluids (e.g., gripe water), and vitamins, minerals, or medicines (e.g., oral rehydration salts, drops, syrups) [23]. Lactation intensity was determined by adapting a tool previously validated and used by our group [24,25]. Mothers in the MMS arm also had to self-report ≥50% adherence with the study-administered MMS.

### Ethics approval

Prior to obtaining informed consent, research personnel verbally explained the purpose and voluntary nature of the study. Ethics approval for this study was obtained from the Aga Khan University (Karachi, Pakistan), Hospital for Sick Children (SickKids; Toronto, Canada), National Bioethics Committee Pakistan (Islamabad, Pakistan), and University of Toronto (Toronto, Canada).

### Data collection

Demographic and birth-related characteristics were obtained from the MaPPS Trial data. Given the low level of literacy in the study population, tablet-based (Samsung Galaxy Tab A T285; Samsung, Vietnam) questionnaires were verbally administered by trained personnel to capture data from participants. This included an assessment of household-level food security over the past month [26]. Our previously described 24-h dietary recall tool [16] was administered, and dietary data were used to generate minimum dietary diversity score [27].

### Anthropometry

Anthropometric measures were collected in duplicate by 2 study personnel, using standardized procedures. Maternal measures included height and weight at 3-mo postpartum [18]. Maternal BMI (in kg/m^2^) was calculated. Standard WHO cut-offs were applied to determine measures of maternal under- and overnutrition (underweight: BMI <18.5; overweight and obese: BMI ≥25.0) [28]. Infant measures included length, weight, head circumference, and middle-upper arm circumference (MUAC) at birth and 3-mo postpartum [18]. The WHO Child Growth Standards were applied to the mean of 2 acceptable measures to generate z-scores by applying the publicly available WHO igrowup package for Stata [29]. Infant nutrition indexes were defined according to the WHO criteria, including z-scores for length-for-age (LAZ; stunting: <–2 SD); weight-for-age (WAZ; underweight: <–2 SD); weight-for-length (WLZ; wasting: <–2 SD); head circumference-for-age (HCZ; microcephaly: <–2 SD); and MUAC-for-age (MUAZ) [30,31].

### Milk sample collection and storage

To standardize milk collection, trained personnel guided mothers on how to collect all milk from 1 breast (fore- and hind-milk) into a sterile polypropylene bottle using hand expression 2 h after the last feeding, and between 10:30 to 12:30 [32]. From the complete expression, ≤20 mL were aliquoted into cryovials, placed into an insulated container with ice packs, and stored out of direct sunlight. Samples were transported to the field site laboratory within an hour and stored at −80°C and then shipped on dry ice to the Hospital for Sick Children for storage at −80°C until analysis (Supplemental Table 3). All mothers were provided with instructions around how to safely store the remaining expressed milk and feed it to their infant.

### Milk nutrient analysis

All milk nutrient analysis was conducted in duplicate, except for the macronutrients and iodine, with the mean of 2 measures used in analyses. Milk fat, true protein, and carbohydrates were evaluated using a mid-infrared analyzer (Miris Human Milk Analyzer, Miris AB®, Uppsala, Sweden; Supplemental Methods). Metabolizable energy was calculated by applying Atwater factors to protein (4 kcal/g), carbohydrates (4 kcal/g), and fat (9 kcal/g).

Iodine was analyzed by inductively coupled plasma mass spectrometry in kinetic energy discrimination mode (iCAP Q, Thermo Fisher Scientific), following the published methods of Huynh et al. (Supplemental Methods) [33]. Samples were quantified against a standard curve with 4 concentrations from 0.1 to 10 μg/L; the average r^2^ of the standard curve was 0.996 with a SD of 0.004 [34]. Two sample values were above the upper limit, but these were maintained given the linearity of the curve. Vitamin A (retinol) and E (α-tocopherol) were analyzed by HPLC with diode array detection (Agilent 1260 Infinity II), similarly to previously described methods (Supplemental Methods) [35]. The lower and upper limit of quantification for retinol were 0.02 to 1.54 μg/mL, and 0.18 to 70.0 μg/mL for α-tocopherol; all sample values were within this range. Fat concentration was used to adjust sample vitamin A and E values, because milk fat is known to influence fat-soluble vitamin composition [36]. Vitamin B12 (cobalamin) was analyzed by competitive chemiluminescent enzyme immunoassay, using the IMMULITE 2000 and IMMULITE 2000 Vitamin B12 Kit (Siemens) via an adapted method for skimmed human milk (Supplemental Methods) [37,38]. The lower and upper limit of quantification were 37 and 738 pmol/L. Twenty-five samples were below 37 pmol/L (mean: 28 pmol/L, range: 13–36 pmol/L), although these values were maintained in the analyses because diluted milk maintained linearity as low as 16 pmol/L (r^2^ = 0.981) [37]. Folate was analyzed by microbiological assay following trienzyme digestion [39], by adopting previously published methods for skimmed milk (Supplemental Methods) [40,41]. The lower and upper limit of quantification were 17 to 333 nmol/L; all sample values were within this range. When analyzing milk folate, sodium ascorbate is typically added as a stabilizer to fresh milk. Although we did not add sodium ascorbate, we found that there was no difference between fresh samples at baseline and after storage for 4 mo at −80°C with or without sodium ascorbate (Supplemental Table 4). Quality controls were analyzed with each batch of samples, and included National Institute of Standards and Technology (NIST) standard reference material corresponding to the micronutrient of interest [NIST 1869a: iodine (coefficient of variation: 9.2%) and vitamin B12 (4.3%); NIST 1950: retinol (10.3%), α-tocopherol (6.2%), and folate (12.2%)] and an in-house pooled milk sample. The inter-assay coefficient of variation for pooled milk samples were 5.7% for iodine, 4.9% for retinol, 8.4% for α-tocopherol, 7.0% for vitamin B12, and 15.0% for folate (Supplemental Table 4).

### Estimated adequate concentration of milk micronutrients

Milk micronutrient measures were compared with corresponding estimated adequate milk concentrations described by the Institute of Medicine [IOM; in 2015, the IOM became a part of the National Academies of Science, Engineering, and Medicine (NASEM)] and WHO. Because there are acknowledged limitations to these concentrations, including generalizability, methodology, and lack of longitudinal data [42], we summarized our data compared to multiple values. For iodine, this included <115 μg/L [43] and <141 μg/L [44]; vitamin A, <0.485 μg/mL [44], <0.3 μg/mL, and <8.0 μg/g fat [45]; vitamin E, <3.2 μg/mL [43] and <4.9 μg/mL [46]; vitamin B12, <310 pmol/L [47] and <295 pmol/L [43]; and folate, <193 pmol/L [47]. We also compared our data to concentrations from studies conducted in comparable settings for iodine sufficiency <231 μg/L [48] and vitamin B12 deficiency <87 pmol/L [49]. We have collectively termed these cut-points “mother’s milk adequacy estimates” (MAEs) throughout this article, given the limitations of the concentrations. We further determined the possible combinations of concurrent milk micronutrients below the IOM MAEs observed among participants, as the IOM milk micronutrient concentrations are commonly used in the literature and several WHO milk micronutrient concentrations are based on the IOM values. We then generated a variable for the number of concurrent milk micronutrients below the IOM MAEs and a categorical variable to compare those with all 5 milk micronutrients below the IOM MAEs to other combinations.

### Maternal blood collection and analysis

At the time of substudy enrolment (i.e., 3-mo postpartum), maternal hemoglobin concentration was assessed via finger prick, using the HemoCue Hb 301 System (HemoCue). The anemia cut-off was applied (hemoglobin <12 g/dL) [50]. Within the MaPPS Trial, maternal venous blood (5 mL) was collected at 1-wk postpartum by trained phlebotomists, as previously described [18]. Select serum micronutrient concentrations were analyzed at the Nutrition Research Lab, Aga Khan University. Serum vitamin A (retinol) was determined by isocratic reversed-phase HPLC (Agilent 1200 series HPLC) with UV-VIS detection at 325 nm using elution through an octadecylsilane column and calculating each analyte’s concentration by internal standard method using retinyl acetate; the upper and lower limit of quantification were 0.02 and 10.5 μmol/L; all sample values were within this range. Sigma Retinol (95144) was used as a standard reference material (coefficient of variation: 1.2%) and ClinChek Serum Control, lyophil for vitamins for quality control. Serum vitamin B12 was determined by electrochemiluminescence immunoassay (Roche Cobas e411 automated immunoanalyzer) using the Roche Elecsys Vitamin B12 II kit; the lower and upper limits of quantification were 37 and 1481 pmol/L; all sample values were within this range. Roche Elecsys PreciControl Varia was used for quality control (Supplemental Table 5) [51,52].

### Sample size

Given the absence of data in the study setting to anticipate the impact of MMS into postpartum on either milk micronutrient concentration, or the association of milk micronutrient concentrations and growth [53], the sample size was set to be able detect an effect size of r = 0.24 between milk nutrients and infant linear growth; assuming 80% power and a significance level of α < 0.05, 194 participants were required [54]. To account for a proportion of subjects being unable to provide a milk sample, we aimed to recruit 220 participants.

### Statistical analysis

All statistical analyses were conducted using Stata version 15 (Stata Corporation, USA). Descriptive statistics were used to summarize maternal and infant participant characteristics, including means with SDs and counts with proportions for continuous and categorical variables, respectively. All continuous outcomes variables were assessed for normality using the Shapiro-Wilk test (Supplemental Table 6). Because all micronutrient concentrations were left-skewed, geometric means and 95% CIs have been presented. Continuous outcomes were compared using linear regression and categorical outcomes using logistic regression. To account for cluster randomization within the MaPPS Trial, robust standard errors were generated in all analyses. Statistical significance was defined as *P* < 0.05. Where data were missing, they were excluded from the analyses.

#### Milk and serum micronutrients by arm

Following transformation, micronutrient concentrations were compared first without adjustment using linear regression. In the adjusted analyses, preidentified variables from the literature (maternal age, parity, maternal BMI category, number of milk feeds in a day) were included and serially removed to investigate the effect on the β-coefficient and potential confounding. Maternal BMI category and milk feeds were retained in all models because of an imbalance between arms. For purposes of simplicity, only adjusted analyses are presented in-text; both unadjusted and adjusted estimates are presented in tables. Regression was also used to investigate the association between vitamin A and B12 concentration in milk collected at 3-mo postpartum and maternal serum collected at 1-wk postpartum. Logistic regression was also used to compare the proportion of samples below the serum cut-offs by arm, including <0.70 μmol/L [55] and <1.05 μmol/L [44] for serum vitamin A and <150 pmol/L for serum vitamin B12 [56]. Because hemoglobin concentration can be influenced by different micronutrient deficiencies (e.g., vitamin A, folate, vitamin B12) [50], regression was also used to compare maternal hemoglobin concentration between mothers with all 5 milk micronutrients below the estimated adequate requirement and without.

#### Infant growth by arm

To compare infant continuous (LAZ, WAZ, WLZ, HCZ) and categorical (stunting, underweight, wasting, microcephaly) growth measures at birth and 3-mo postpartum, a repeated measures analysis using generalized estimating equations was performed, including main effects for intervention and time and an interaction term for intervention and time. MUAZ cannot be calculated at birth using WHO Anthro, thus measures at 3-mo postpartum were compared using linear regression. Crude and adjusted analyses have been presented using the adjustments described above.

#### Exploring associations between infant anthropometry and each milk micronutrient

Associations between each milk micronutrient concentration and each infant anthropometric measure (LAZ, WAZ, WLZ, HCZ, and MUAZ) at 3-mo postpartum were conducted on the combined dataset irrespective of arm. We then investigated associations between infant anthropometric measures and each IOM MAE, as the IOM values are more common. We also explored the association between each infant anthropometric measures in relation to the categorical variable for having all 5 milk micronutrients below the MAEs and the continuous variable for the number of concurrent milk micronutrients below the MAEs. Analyses were completed using linear regression, with and without adjustment, as previously described. In all cases, the anthropometric measure was the dependent variable.

## Results

**FIGURE 1:**
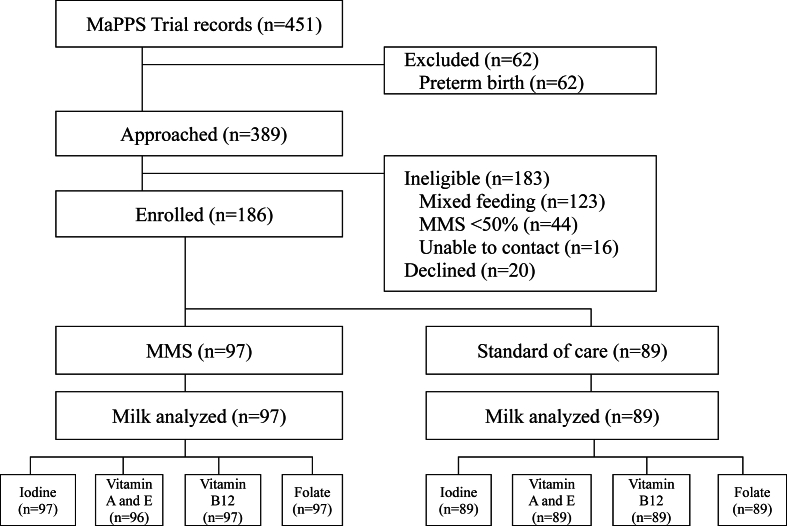
Participant flow diagram from record review to enrolment and milk sample analysis. MaPPS Trial, Matiari emPowerment and Preconception Supplementation Trial; MMS, multiple micronutrient supplement.

Upon review of the MaPPS Trial records in August 2020, 451 infants 3 mo of age or less were identified to be enrolled in the trial, and 389 were term-born (Figure 1). Of the 389 mother-infant dyads approached for participation, 186 enrolled in the substudy (MMS: *n* = 97, standard of care: *n* = 89; Figure 1) from August 2020 to April 2021. Reasons for ineligibility included mixed feeding (*n* = 123), <50% MMS adherence (*n* = 44), and inability to achieve contact due to temporary migration (*n* = 16), as well 20 dyads declined participation. The target sample size was not achieved because the MaPPS Trial was nearing completion, and the rate of mixed feeding was higher than anticipated in the area.

On average, participants were primiparous mothers aged 23 y to infants aged 3 mo (Table 1) [26]. Fourteen participants (7.5%) were <19 y of age at enrolment. Demographic characteristics were largely comparable between arms, although among mothers receiving MMS more were underweight (28% vs. 19%) and fewer were overweight or obese (8% vs. 24%). Maternal diets consisted primarily of micronutrient-limited staple foods, with 82% below the cut-off for minimum dietary diversity score (<5 food groups). Few infants were exclusively breastfed in either arm (15%), although nearly all feeds in a day to study infants were milk (88%). Among predominantly breastfed infants (85%), 89% had only water or water and/or 1 nonnutritive feed (e.g., gripe water) in a day. Infants in the standard of care arm were fed at the breast once more a day than in the MMS arm (10 vs. 11 times per day).

**TABLE 1:** Maternal, infant, and household characteristics among study participants (*n* = 186 dyads).

Characteristic	Value^1^		Standardized difference
	MMS (*n* = 97)	Standard of care (*n* = 89)	
Maternal characteristics			
Maternal age (y)	22.5 ± 2.1	22.7 ± 2.2	0.12
Primiparous	58 (59.8)	57 (64.0)	−0.09
Maternal education			0.09
None	58 (59.8)	48 (53.9)	
Primary	23 (23.7)	25 (28.1)	
Secondary or higher	16 (16.5)	16 (18.0)	
BMI (kg/m^2^)	20.8 ± 3.3	21.7 ± 3.8	0.28
BMI category			0.39
<18.5 kg/m^2^ (underweight)	27 (27.8)	17 (19.1)	
18.5–24.9 kg/m^2^ (normal)	62 (63.9)	51 (57.3)	
≥25 kg/m^2^ (overweight or obese)^2^	8 (8.2)	21 (23.6)	
Dietary diversity score	3.3 ± 1.2^3^	3.3 ± 1.3^3^	0.03
Hemoglobin concentration (g/dL)^4^	12.0 ± 1.1	12.2 ± 1.5	0.14
Anemic (<12 g/dL)	46 (47.4)	36 (41.4)	0.12
Infant characteristics			
Sex (female)	44 (45)	34 (38)	−0.14
Low birthweight (<2500 g)	32 (33)	34 (38)	0.11
Small-for-gestational age at birth	50 (52)	49 (54)	0.05
Age at study enrolment (mo)	3.1 ± 0.5	3.1 ± 0.4	0.04
Lactation status			−0.11
Exclusive	17 (18)	12 (13)	
Predominant	80 (82)	77 (87)	
Total number of feeds in a day	11.6 ± 2.6	12.2 ± 2.3	0.26
Number of milk feeds in a day	10.0 ± 2.2	10.9 ± 2.2	0.39
Proportion of human milk feeds^5^	89%	87%	0.25
Household characteristics			
Wealth quintile			0.03
Q1 (poorest)	21 (21.6)	22 (24.7)	
Q2	22 (22.7)	18 (20.2)	
Q3	27 (27.8)	18 (20.2)	
Q4	18 (18.6)	22 (24.7)	
Q5 (least poor)	9 (9.3)	9 (10.1)	
Household food security^6^			−0.10
No food insecurity	86 (88.7)	77 (86.5)	
Mild food insecurity	6 (6.7)	3 (3.1)	
Moderate food insecurity	5 (5.6)	7 (7.2)	
Severe food insecurity	0 (0.0)	2 (2.1)	

MMS, multiple micronutrient supplement.

1 Values are mean ± SD or n (%).

2 2 mothers with obesity (1 per study arm), thus categories of obese and overweight were combined;

3 Range of dietary diversity scores in MMS and standard of care arms: 1 to 6.

4 2 mothers elected not to complete HemoCue assessment (*n* = 184).

5 Proportion of human milk feeds was calculated for each infant by dividing the number of human milk feeds in a day by the total number of feeds in a day (e.g., human milk. water, rice water), and multiplying by 100%.

6 Food security was determined using the Household Food Insecurity Access Scale [26].

### Milk nutrient content by study arm and maternal serum values

The iodine concentration and retinol per gram fat were higher among those supplemented with MMS compared with the standard of care [geometric mean (95% CI): 122 (104, 141) compared with 77 (67, 89) μg/L and 7.1 (6.3, 8.0) compared with 5.6 (4.8, 6.6) μg/g fat, respectively; Table 2]. There was no difference between arms for α-tocopherol, folate, or vitamin B12. There was no difference in maternal serum retinol and vitamin B12 between arms, although there was a positive association between maternal serum collected at 1-wk and milk collected at 3-mo postpartum for both vitamin B12 (β: 0.38, 95% CI: 0.23, 0.53) and retinol (β: 0.05, 95% CI: 0.05, 0.09) concentration.

**TABLE 2:** Nutritional characteristics of milk samples collected at 3-mo postpartum and maternal serum collected at 1-wk postpartum.

Characteristic	Value^1^		*P* value	
	MMS (*n* = 97)	Standard of care (*n* = 89)	Crude	Adjusted^2^
Human milk collected at 3-mo postpartum				
Volume of expression, mL	58.7 ± 26.3	56.8 ± 24.7	0.60	0.49
Fat, g/dL	4.1 (3.8, 4.4)	4.1 (3.8, 4.6)	0.50	0.72
True protein, g/dL	0.92 (0.89, 0.96)	0.96 (0.92, 0.99)	0.21	0.23
Carbohydrates, g/dL	8.1 (8.0, 8.1)	8.1 (8.0, 8.2)	0.36	0.48
Metabolizable energy, kcal/dL	75.1 (72.3, 78.1)	76.5 (73.0, 80.2)	0.42	0.62
Iodine, μg/L	122 (104, 142)	77 (67, 89)	<0.0001	<0.0001
Retinol, μg/mL^3^	0.29 (0.25, 0.33)	0.24 (0.20, 0.28)	0.12	0.09
Retinol, μg/g fat^3^	7.1 (6.3, 8.0)	5.6 (4.8, 6.6)	0.04	0.04
α-tocopherol, μg/mL^3^	3.2 (2.9, 3.5)	3.3 (2.9, 3.6)	0.70	0.51
α-tocopherol, μg/g fat^3^	77.6 (72.3, 83.3)	78.0 (72.0, 84.4)	0.93	0.49
Vitamin B12, pmol/L	78 (69, 88)	71 (60, 84)	0.37	0.36
Folate, nmol/L	61 (57, 66)	56 (53, 60)	0.08	0.15
Maternal serum collected at 1-wk postpartum				
Retinol, μmol/L^4^	0.60 (0.52, 0.69)	0.68 (0.60, 0.78)	0.17	0.34
Vitamin B12, pmol/L^5^	190 (165, 220)	201 (172, 235)	0.62	0.56

MMS, multiple micronutrient supplement.

1 Results are geometric means (95% CIs).

2 Adjusted for maternal BMI category and number of human milk feeds in a day.

3 *N*_*MMS*_ = 96.

4 *N* = 175 (MMS: 94; standard of care: 81).

5 *N* =163 (MMS: 87; standard of care: 76).

Most milk samples were below the MAEs for all micronutrients regardless of arm (Table 3). Fewer milk samples in the MMS arm compared with the standard of care were below the IOM MAE for iodine (MMS: 60%, standard of care: 81%; *P* = 0.004) and the WHO MAE for retinol after adjusting for fat (MMS: 52%, standard of care: 67%; *P* = 0.02). There was no difference in the number of samples below the MAEs for α-tocopherol and vitamin B12. All samples were below the MAE for folate. A similar trend was observed for maternal serum retinol and vitamin B12 (Table 3). Mothers with milk vitamin B12 below the <87 pmol/L cut-point who received MMS had higher milk vitamin B12 than those who received standard of care [55 (50, 60) pmol/L compared with 44 (39, 50) pmol/L; *P* = 0.007]. Mothers identified as vitamin B12 deficient using serum who received MMS had higher milk B12 than those who received standard of care [68 (58, 79) pmol/L compared with 50 (36, 69) pmol/L], albeit marginally (*P* = 0.07).

**TABLE 3:** Proportion of milk samples collected at 3-mo postpartum and maternal serum samples collected at 1-wk postpartum with micronutrient values below concentrations indicative of inadequacy.

Micronutrient	n (%)		*P* value	
	MMS (*n* = 97)	Standard of care (*n* = 89)	Crude	Adjusted^1^
Human milk collected at 3-mo postpartum				
Iodine				
Below IOM^2^ concentration (<141 μg/L)	58 (60)	71 (81)	0.004	0.004
Below WHO concentration (<115 μg/L)	52 (54)	58 (66)	0.11	0.09
Below WHO adequacy concentration (<231 μg/L)	80 (82)	85 (97)	0.009	0.01
Retinol^3^				
Below IOM^2^ concentration (<0.485 μg/mL)	70 (73)	72 (81)	0.20	0.15
Below WHO deficiency concentration (<0.3 μg/mL)	48 (50)	49 (55)	0.49	0.34
Below WHO deficiency concentration (<8.0 μg/g fat)	50 (52)	60 (67)	0.03	0.02
α-tocopherol^3^				
Below IOM^2^ concentration (<4.9 μg/mL)	76 (79)	69 (78)	0.79	0.76
Below WHO concentration (<3.2 μg/mL)	48 (50)	38 (43)	0.32	0.32
Vitamin B12				
Below IOM^2^ concentration (<310 pmol/L)	93 (96)	83 (93)	0.43	0.43
Below WHO concentration (<295 pmol/L)	93 (96)	83 (93)	0.43	0.43
Below deficiency concentration (<87 pmol/L)	61 (63)	53 (60)	0.64	0.81
Folate				
Below IOM^2^ and WHO concentration (<193 nmol/L)	97 (100)	89 (100)	-	-
Maternal serum 1-wk postpartum				
Retinol^4^				
Below IOM^2^ concentration (<1.05 μmol/L)	77 (82)	63 (78)	0.50	0.69
Below WHO concentration for populations (<0.70 μmol/L)	51 (54)	39 (48)	0.42	0.54
Vitamin B12^5^				
Below IOM^2^ and WHO concentration (<150 pmol/L)	35 (37)	27 (33)	0.54	0.52

MMS, multiple micronutrient supplement; IOM, Institute of Medicine.

1 Adjusted for maternal BMI category and number of human milk feeds in a day.

2 Now referred to as NASEM.

3 *N*_*MMS*_ = 96.

4 *N* = 175 (MMS: 94; standard of care: 81)

5 *N* = 163 (MMS: 87; standard of care: 76)

Using the IOM MAEs, 13 different combinations of low milk micronutrient concentrations were observed among participants (Supplemental Table 7). The distribution did not differ between the arms (*P* = 0.18). Forty-six percent of participants had milk micronutrient concentrations below the MAE for all micronutrients investigated (MMS: 38%, standard of care: 54%; *P* = 0.03). Although no difference in maternal hemoglobin concentration was observed between arms (Table 1), the average hemoglobin concentration for those with all milk micronutrient concentrations below the MAEs was marginally lower [11.9 (11.6, 12.2) vs. 12.3 (12.0, 12.5) g/dL; *P* = 0.06, *n* = 184].

### Infant growth and milk micronutrient composition

There was no difference between arms for any of the infant growth outcomes considered (Table 4). Exploratory analyses found no statistically significant associations between individual milk micronutrient concentration and any anthropometric measures at 3 mo, with the exception of α-tocopherol concentration and LAZ (β: 0.10, 95% CI: 0.009, 0.20, *P* = 0.03; Supplemental Table 8). Among infants who had consumed milk with concentrations below the MAEs for retinol and α-tocopherol, a decrease in LAZ was observed (Table 5). No other association was observed for the other milk micronutrient concentrations below the MAEs and anthropometric outcomes (Supplemental Table 8). However, infants consuming milk with all 5 milk micronutrients below the MAEs were found to have significantly lower LAZ (β: −0.39, 95% CI: −0.73, −0.04; *P* = 0.03) and a marginally lower HCZ (β: −0.32, 95% CI: −0.63, 0.001; *P* = 0.05; Table 5) than those that did not. The effect size increased proportionally with the number of milk micronutrients below the MAEs for LAZ, but not HCZ (Supplemental Table 8). This association was not observed for WAZ, WLZ, or MUAC (Supplemental Table 8).

**TABLE 4:** Infant anthropometric characteristics at birth and 3-mo postpartum (*n* = 186).

Characteristic	Value^1^		*P* value		
	MMS (*n* = 97)	Standard of care (*n* = 89)	Treatment effect	Time effect	Treatment and time interaction
Continuous growth measures					
Length-for-age z-score					
Birth	−1.00 (−1.24, −0.75)	−1.14 (−1.45, −0.84)	0.46	0.001	0.88
3-mo postpartum	−1.42 (−1.67, −1.17)	−1.59 (−1.83, −1.35)			
Weight-for-age z-score					
Birth	−1.26 (−1.48, −1.03)	−1.30 (−1.57, −1.04)	0.79	0.002	0.91
3-mo postpartum	−1.68 (−1.92, −1.44)	−1.71 (−1.96, −1.46)			
Weight-for-length z-score					
Birth^2^	−1.08 (−1.33, −0.83)	−0.96 (−1.23, −0.69)	0.51	0.009	0.87
3-mo postpartum	−0.72 (−0.96, −0.48)	−0.57 (−0.84, −0.29)			
Head circumference-for-age z-score					
Birth	−0.82 (−1.07, −0.56)	−0.82 (−1.11, −0.52)	0.99	0.08	0.95
3-mo postpartum	−1.04 (−1.27, −0.81)	−1.06 (−1.28, −0.84)			
Middle-upper arm-for-age z-score^3^					
3-mo postpartum	−1.21 (−1.48, −0.93)	−0.95 (−1.23, −0.68)	0.19	-	-
Categorical growth measures					
Stunting					
Birth	17 (18)	24 (27)	0.18	0.06	0.56
3-mo postpartum	30 (31)	34 (38)			
Underweight					
Birth	25 (26)	30 (34)	0.24	0.20	0.60
3-mo postpartum	36 (37)	38 (43)			
Wasting					
Birth^2^	20 (22)	17 (22)	0.90	0.23	0.91
3-mo postpartum	14 (14)	13 (15)			
Macrocephaly					
Birth	15 (15)	16 (18)	0.51	0.40	0.11
3-mo postpartum	24 (25)	15 (17)			

MMS, multiple micronutrient supplement.

1 Values for continuous growth measures are mean (95% CI) and *N* (%) for categorical growth measures.

2 WLZ and wasting at birth could not be determined for 16 infants because their length at birth was <45 cm, which falls outside of the range of the WHO Anthro software (6 in the MMS arm and 10 in the standard of care arm).

3 WHO Anthro software does not allow the application of the MUAC growth curves at birth.

**TABLE 5:** Associations between selected infant anthropometric measures at 3-mo postpartum and the number of milk micronutrient concentrations below the IOM MAEs using all participant data (*n* = 186) in the exploratory analyses.

Characteristics	N	β (95% CI)	Crude *P* value	β (95% CI)	Adjusted *P* value^1^
Length-for-age z-score (LAZ)					
Individual MAEs^2^					
Iodine <141 μg/L	129	−0.005 (−0.40, 0.39)	0.98	−0.04 (−0.44, 0.36)	0.84
Retinol <0.485 μg/mL	142	−0.40 (−0.82, 0.01)	0.06	−0.39 (−0.82, 0.04)	0.08
Retinol <8.0 μg/g fat	97	−0.33 (−0.67, 0.02)	0.06	−0.32 (−0.66, 0.03)	0.08
α-tocopherol <4.9 μg/mL	145	−0.62 (−1.00, −0.22)	0.002	−0.62 (−1.02, −0.22)	0.003
Below MAEs for all 5 milk micronutrients (IOM)^3^	85	−0.37 (−0.71, −0.03)	0.03	−0.39 (−0.73, 0.04)	0.03
Below MAEs for all 5 milk micronutrients (mix)^4^	63	−0.49 (−0.85, −0.13)	0.008	−0.49 (−0.85, −0.13)	0.008
Head circumference-for-age z-score (HCZ)					
Individual MAEs^2^					
Iodine <141 μg/L	129	0.15 (−0.19, 0.50)	0.38	0.08 (−0.28, 0.44)	0.65
Retinol <0.485 μg/mL	142	−0.33 (−0.72, 0.07)	0.11	−0.30 (−0.70, 0.10)	0.14
Retinol <8.0 μg/g fat	97	−0.09 (−0.42, 0.23)	0.57	−0.07 (−0.39, 0.25)	0.66
α-tocopherol <4.9 μg/mL	145	−0.33 (−0.70, 0.04)	0.08	−0.32 (−0.70, 0.05)	0.09
Below MAEs for all 5 milk micronutrients (IOM)^3^	85	−0.29 (−0.61, 0.03)	0.08	−0.32 (−0.63, 0.001)	0.05
Below MAEs for all 5 milk micronutrients (mix)^4^	63	−0.37 (−0.70, −0.04)	0.03	−0.37 (−0.69, −0.05)	0.02

MAE, mother’s milk adequacy estimate.

1 Adjusted for maternal BMI category and number of human milk feeds in a day.

2 Data not presented for vitamin B12 and folate because ≥95% of participants were below the corresponding MAEs.

3 Categorized as “below MAEs for all 5 milk micronutrients (IOM)” if iodine <141 μg/L, vitamin A <0.485 μg/mL, vitamin E <4.9 μg/mL, vitamin B12 <310 pmol/L, and folate <193 nmol/L).

4 Categorized as “below MAEs for all 5 milk micronutrients (mix)” if iodine <141 μg/L, vitamin A <8.0 μg/g fat, vitamin E <4.9 μg/mL, vitamin B12 <310 pmol/L, and folate <193 nmol/L).

## Discussion

To our knowledge, this is the first study to investigate mature milk micronutrient composition following continued MMS supplementation (UNIMMAP formulation from preconception to lactation), compared with standard of care (iron and folic acid supplementation during pregnancy and lactation). Although we found differences between arms in milk iodine concentration and retinol per gram fat, most samples in both arms did not meet existing MAEs for micronutrients of interest. Infant growth did not differ between arms, but infants fed milk with all 5 micronutrients below MAEs had decreased LAZ and HCZ.

Our observations that MMS compared with standard of care increased milk iodine concentration and retinol per gram fat, but not vitamin B12, α-tocopherol, or folate concentration, likely reflect differences in supplement composition; dietary intake from nonsupplemental sources in the region; micronutrient bioavailability; and mechanisms of milk secretion. Milk samples could be low in other micronutrients important to growth (e.g., zinc); however, analyzed micronutrients were purposively selected given regional maternal deficiency, supplement composition, and role in development. Milk sample macronutrients were within normal ranges [[57], [58], [59]].

Although milk iodine increased with MMS, 60% of mothers’ milk iodine remained <141 μg/L (IOM MAE). Our findings are consistent with Dror and Allen [10], wherein high or daily iodine supplementation increased milk concentration. In Pakistan, 45.6% of females experience urinary iodine <100 μg/L, 17.9% moderate or severe iodine deficiency, and 7.7% goiter [13]. The study region is inland with iodine deplete soil, making MMS an important dietary iodine source. We observed milk iodine concentrations similar to observational studies and/or iodine supplementation trials from LMICs [[60], [61], [62]]. Given iodine’s role in thyroid hormone production, our observed difference in milk iodine may impact early infant neurodevelopment.

Despite an observed increase in milk retinol per gram fat, 52% of MMS samples had milk concentrations <8.0 μg retinol per gram fat compared to 67% of standard of care. Retinol is sequestered into the mammary gland over maternal reserves [9], and we observed a positive association between maternal serum and milk retinol, as reported by others [9,63,64]. Using serum values, vitamin A deficiency among study lactating mothers was higher than regional estimates (51% vs. 30%) [13]. Relative to other LMIC-based studies, our milk retinol concentrations were similar [[65], [66], [67], [68]], or lower [7, [69], [70], [71], [72]], than in observational studies; net-differences align with similar vitamin A supplementation studies [67,68]. Given vitamin A plays an important role in immunity, cell growth, and differentiation, our observations have potential implications for early infant morbidity, growth, and development.

We did not observe a difference in milk vitamin B12 between groups, although 38% of lactating participants were deficient using serum concentrations. The relationship between maternal vitamin B12 stores and milk is poorly understood; when maternal stores are low, which the body prioritizes is not clear [11]. Employing the <87 pmol/L milk vitamin B12 cut-point, per Duggan et al. [49], most (61%) participants’ values were below. Vitamin B12 concentrations we observed were within mature milk ranges reported by other LMICs-based studies [37,49,[73], [74], [75], [76]]. Young et al. [77] found that milk vitamin B12 concentration at 2-wk postpartum (transitional milk) in Pakistan was higher without or with supplementation until birth (average: 485 pmol/L) than the mature milk in our study. That few participants in our study achieved the vitamin B12 MAEs is consistent with LMIC-based studies providing a comparable supplemental dose [7,37,71,[74], [75], [76],^78^].

Of note, an Indian study of 6 hospitalized infants fed human milk with vitamin B12 ∼32.5 pmol/L experienced severe neurological and developmental symptoms, which were corrected with direct vitamin B12 supplementation [79]. In our study, 18 infants’ milk vitamin B12 concentrations were <32.5 pmol/L (4 and 14 receiving MMS and standard of care, respectively; *P* = 0.007). Given vitamin B12 is essential for DNA synthesis and myelination [12], and evidence linking low milk vitamin B12 to neurological sequelae, future studies are warranted to assess any clinical implications of our findings.

We found no difference in milk folate or vitamin E concentration between arms. All samples were below the folate MAE. Milk folate secretion is generally under tight homeostatic control and unaffected by supplementation at the provided dose, except in cases of extreme folate deficiency presenting as megaloblastic anemia [1,80]. National data from Pakistan suggest half of reproductive-age females are folate deficient (<10 nmol/L) using serum; this cut-point is associated with megaloblastic anemia risk [15]. Of note, a higher preconception serum folate cut-point (28–30 nmol/L) has been recommended clinically to reduce risk of a pregnancy affected by neural tube defects [81]. For vitamin E, our concentration was comparable to other studies of α-tocopherol in LMICs [7,66,67,71,82] and a recently published meta-analysis, suggesting mature milk α-tocopherol concentration was 3.3 μg/mL (95% CI: 3.0, 3.6; 42 studies) [83]. Although vitamin E is suggested to play a potential role in immunity, an association between α-tocopherol and infant growth has not been documented [53], and little is known about vitamin E status across the lifespan in Pakistan.

At birth, 53% of study infants were classified as small-for-gestational age, likely due to intrauterine growth restriction related to maternal malnutrition. At 3-mo postpartum, 34% of study infants were classified as stunted. Infants fed milk wherein all micronutrients were below corresponding MAEs had lower LAZ and HCZ, with a sizeable magnitude of difference. We likely lacked the distribution required to see relationships between individual milk micronutrient concentrations and infant growth measures due to high regional undernutrition and our sample size.

Strengths of our study included systematically enrolling infants of similar age (term-born, 3-mo postpartum) and lactation profile (no reported formula or mixed feeding); collecting and storing representative samples per best practices (full breast expression collected late-morning, stored at −80°C within 1 h) [32]; and processing samples using current milk methodologies [84]. Including multiple samples over time and intake volume would have further informed infants’ actual micronutrient intake. Seasonal effects on dietary intake were not elucidated, and maternal serum and milk sample collection was not concurrent. As the MaPPS Trial was an effectiveness study, we do not have detailed MMS adherence data. Using pill count data from quarterly postpartum monitoring visits (*n* = 75), MMS adherence was 67%. We also did not measure all possible milk or infant factors that relate to growth (e.g., human milk oligosaccharides, zinc, infant morbidity) [53,85], and did not adjust for multiple comparisons (e.g., Bonferroni correction). We did not achieve our sample size, and estimate being underpowered to examine an effect of MMS on vitamin A and B12. Our effect estimates could be useful in justifying future studies. With a larger sample size, micronutrient-growth associations may be observed. Nonetheless, considering milk micronutrients collectively was an analytic strength, as micronutrients work in tandem to support growth and development.

Importantly, there are acknowledged limitations to MAEs, including development from few studies of small size and restricted generalizability, and issues related to systematic sample collection, methodology, lack of validation, and variability (e.g., maternal status, lactation stage) [42]. Furthermore, consideration of long-term outcomes (e.g., growth faltering, neurodevelopmental delay) is lacking [53]. Among females in areas with low micronutrient intakes, debate exists whether MAEs are higher than necessary to support adequate infant growth and development, highlighting the need for better understanding. Anticipated reference values generated via the Mothers, Infants, and Lactation Quality (MILQ) Study will be informative [86].

In conclusion, in a rural Pakistani setting with dietary limitations and micronutrient deficiencies, providing maternal MMS at UNIMMAP formulation may not provide maternal nutrition sufficient to produce milk micronutrient values above MAEs, and may require additional intervention [87]. Our findings could question the use of a universal MMS formulation to adequately meet micronutrient requirements where undernutrition is common (e.g., South Asia). Further investigation of different settings is needed, and future research on growth and neurodevelopment (e.g., cognitive ability, motor development) should be a priority to understand any longer-term implications of low micronutrient intake from milk in this setting. As acknowledged by the Government of Pakistan [88], scaling up existing nutrition strategies to protect mothers and infants is necessary, including supporting lactation, implementing multi-sectoral and multi-pronged approaches, and addressing root causes of malnutrition (i.e., poverty), as is underway within the income support program [89].

## Author contributions

The authors’ responsibilities were as follows – JABB, YW, AID, SA, SS, RB, ZAB, and DLO designed research; YW, KB, JI, SS, and ZAB conducted research; AH provided database support; JABB, KB, JI, SA, MRB, LD, AG, and CH conducted laboratory analysis; JABB performed statistical analysis and wrote first manuscript draft; all authors provided input and contributed to editing the manuscript; DLO and ZAB had primary responsibility for final content and all authors: read and approved the final manuscript.

## Data availability

Data described in the manuscript, code book, and analytic code may be made available upon request pending application to and approval by the primary investigators and institutional review boards.

## Funding

This study was supported by The Centre for Global Child Health Catalyst Grant, Toronto, Canada. JABB received salary support from the Canadian Institutes of Health Research Fellowship Program. The MaPPS Trial was funded by The Bill and Melinda Gates Foundation (OPP1148892) and World Food Programme (HQ15NF493 – CTR). The funders had no involvement in the study design, data collection, analysis and interpretation of the data, writing of the report, or decision to submit the manuscript for publication.

## Conflict of interest

The authors have no conflicts of interest to declare.
