# Supplementary material for: Maternal multiple micronutrient supplementation in rural Pakistan increased some milk micronutrient concentrations, but not infant growth, at three-months postpartum: a randomized controlled trial substudy
Source: Am J Clin Nutr. 2025 May 21;122(1):174–84. doi: 10.1016/j.ajcnut.2025.05.019 (PMC12308085; doi:10.1016/j.ajcnut.2025.05.019)
Supplement: Multimedia component 1 [file mmc1.docx]

**Supplemental Materials**

Title: *Maternal multiple micronutrient supplementation in rural Pakistan increased some milk micronutrient concentrations, but not infant growth, at three-months postpartum: a RCT substudy*

First author: Baxter, JB

*Supplement composition*

**Supplemental Table 1.** Supplementation regimen by group within the MaPPS Trial, disaggregated by study phase^1^

| **Study phase** | **Intervention** | **Standard of care** |
| --- | --- | --- |
| Preconception | 1 MMS tablet^2^ taken twice in a week | No supplementation |
| Pregnancy | 1 MMS tablet^2^ taken daily upon confirmation of pregnancy (provided by trial) | 1 IFA^3^ tablet taken daily upon confirmation of pregnancy (provided by public health system) |
| Postpartum | 1 MMS tablet^2^ taken daily until 6-months postpartum (provided by trial) | 1 IFA^3^ tablet taken daily until 6-months postpartum (provided by public health system) |

^1^MMS, multiple micronutrient supplement; IFA, iron folic acid

^2^MMS tablets were United Nations International Multiple Micronutrient Antenatal Preparation (UNIMMAP composition; see below)

^3^IFA tablets were per WHO specifications (see below)

**Supplemental Table 2.** MaPPS Trial supplement compositions^1^

| **Micronutrient** | **Multiple Micronutrient Supplement** (UNIMMAP) | | **Iron and folic acid** | |
| --- | --- | --- | --- | --- |
|  | Form | Amount | Form | Amount |
| Vitamins |  |  |  |  |
| Vitamin A | Retinyl acetate | 800 RE | - | - |
| Vitamin B1 | Thiamine nitrate | 1.4 mg | - | - |
| Vitamin B2 | Riboflavin | 1.4 mg | - | - |
| Vitamin B3 | Nicotinamide | 18 mg | - | - |
| Vitamin B6 | Pyridoxine hydrochloride | 1.9 μg | - | - |
| Vitamin B9 (folate) | Folic acid | 400 μg | Folic acid | 400 μg |
| Vitamin B12 | Cyanocobalamin | 2.6 μg | - | - |
| Vitamin C | Ascorbic acid | 70 mg | - | - |
| Vitamin D | Cholecalciferol | 200 IU | - | - |
| Vitamin E | dl-α-tocopheryl acetate | 10 mg TE | - | - |
| Minerals |  |  |  |  |
| Copper | Copper sulphate | 2 mg | - | - |
| Iodine | Potassium iodide | 150 μg | - | - |
| Iron | Ferrous fumarate | 30 mg | Ferrous sulphate | 60 mg |
| Selenium | Selenium selenite | 65 μg | - | - |
| Zinc | Zinc sulphate | 15 mg | - | - |

^1^MaPPS Trial, Matiari emPowerment and Preconception Supplementation Trial; United Nations International Multiple Micronutrient Antenatal Preparation, UNIMMAP

*Human milk compositional analysis*

**Supplemental Table 3.** Timing of human milk compositional analysis

| **Nutrient** | **Timing of Analysis** |
| --- | --- |
| Macronutrients (Miris Human Milk Analyzer) | August – October 2021 |
| Vitamin B12 (cyanocobalamin) | February – April 2022 |
| Vitamin A (retinol) | December 2022 – March 2023 |
| Vitamin E (α-tocopherol) | December 2022 – March 2023 |
| Iodine | February – March 2023 |
| Folate | August – December 2023 |

Macronutrients

Duplicate runs (2x1.5 mL) were conducted on 25% of milk samples (n=47), selected using a random number generator. Because the %CV between duplicate runs was low (i.e., ≤3%; Supplemental Table 3), single runs were completed on the remaining samples to conserve aliquots (n=139).

Characteristics of duplicate runs performed on Miris Human Milk Analyzer 25% of study samples (n=47)^1^

| **Macronutrient** | **Mean difference between runs** | **%CV** |
| --- | --- | --- |
| Fat | -0.1 | 2.2 |
| True protein | -0.1 | 3.0 |
| Carbohydrates | 0 | 2.9 |

^1^CV, coefficient of variation

Iodine

Frozen human milk samples were thawed and mixed by hand. 150 µL of sample was digested in 2.1 mL of 7% tetramethylammonium hydroxide (Sigma-Aldrich, 87741) in a polypropylene tube, vortexed and incubated at room temperature overnight. In the same tubes, the samples were then incubated at 90°C for one hour in a dry block heater, vortexed twice and then diluted to 15 mL with water and triton-X (BioXtra, Sigma-Aldrich, T9284) to a final concentration of 0.05%. Samples were then syringe filtered (Acrodisc 0.2µm, Pall Corporation) into a new polypropylene tube for analysis. All tips and tubes were acid washed in 2% trace grade nitric acid (OnmiTrace, Supelco, VWR CANX0407) overnight, rinsed three times in water and dried in a laminar flow hood. High purity water (Milli-Q, 18.2 MΩ·cm resistivity) was used for all reagents and washing. Precision was assessed with an in-house pools of donor human milk. Accuracy was assessed using nutritional formula standard reference material (1869a, National Institute of Standards and Technology.

Vitamin A and E

Frozen human milk samples (2 mL aliquots) were protected from light and thawed in a 37°C water bath for 30 minutes, vortexed, and sonicated for 7 seconds at 1.5mL/second. 100 µL of sample then underwent saponification and extraction, following the method described by Turner et al (2012). δ-tocopherol was used as an internal standard. Samples were reconstituted with 100 µL of mobile phase (7:2:1 acetonitrile:dichloromethane:methanol), vortexed for 15 seconds, and run at 0.5 mL/min on an Agilent 1260 Infinity II system with UV detection (295 nm: α-tocopherol; 330 nm: retinol), using an injection volume of 50 µL. An InfinityLab Poroshell 120 EC-C18 column (4.6x100mm 2.7μm) and guard column (4.6x5mm 2.7μm) were used. On each sample analysis day, NIST-certified serum (Standard Reference Material 1950) and an in-house pool of donor human milk were extracted and analyzed to assess accuracy and precision, respectively. All samples were run in duplicate.

Vitamin B12

Frozen human milk samples were protected from light and thawed overnight in a refrigerator at 4°C, transferred to a 37°C water bath for 10 minutes (until all fat was immiscible) the morning of analysis and vortexed. 500 μL were transferred to a separate cryovial, and centrifuged at 500 x g for 10 minutes at 4°C. Excess sample was returned to -80°C and used for iodine analysis at a later date. Following centrifugation, fat was removed with a spatula and the supernatant was analyzed on the IMMULITE 2000 in calibration verification mode following the manufacturer specifications. On each sample analysis day, an in-house pool of donor milk was analyzed to assess precision. All samples were run in duplicate.

Folate

Frozen human milk samples were protected from light and transferred to a 37°C water bath for 10 minutes, vortexed, and centrifuged at 500 x g for 10 minutes at 5°C. Fat was removed with a spatula and the supernatant underwent trienzyme digestion, following the method developed by Hyun and Tamura (2005). Following the trienzyme digestion, samples were centrifuged to separate the supernatant, which was aliquoted and stored at -80°C. Folate concentrations were determined within 1-week by a microbial assay using the test organism *Lactobacillus rhamnosus* (ATCC7649; American Type Tissue Culture Collection) and 5-methyltetrahydrofolate to generate the standard curve. On each sample analysis day, an in-house pool of donor milk was analyzed to assess precision. To assess accuracy of the digestion and microbial assay, we analyzed NIST-certified pig liver (Standard Reference Material BCR487) and plasma (Standard Reference Material 1950), respectively. All digested samples were run in duplicate.

*Evaluating folate stability*

Because we did not add a stabilizer to the human milk samples (e.g., sodium ascorbate), as is often done when analyzing human milk folate concentration, we investigated the stability of folate in human milk samples (1) with and without stabilizer and (2) over time at different freezer temperatures. We used excess volumes of recently collected expressed milk samples from 10 participants enrolled in an ongoing study (MaxiMoM: Individualized Fortification of Human Milk for Infants Born ≤ 1250 g; a Three Arm Randomized Clinical Trial; ClinicalTrials.Gov: NCT05308134).

Samples were eligible for inclusion in the stability study if >8 mL of excess milk remained after standard MaxiMoM milk processing procedures. Excess milk was protected from light and stored overnight in a refrigerator, and then warmed in a water bath at 37°C for 30 minutes and protected from light with tinfoil. Samples were then inverted for 1 minute using a SpeciMix (Thermolyne), and 1.2 mL aliquots were placed into cryovials. Aliquots were further treated as described below.

- Fresh samples (baseline)

After defatting 1.2 mL of each fresh sample, samples were digested as previously described. Digested samples were stored at -80°C immediately following digestion, and analyzed via microbial assay the next date.

- Storage for 4 months at -20°C

One aliquot containing whole milk was frozen as is at -20°C in an opaque storage box, and one aliquot was frozen after adding 1% w/v sodium ascorbate and mixing for 15 seconds using a vortex. After 4 months, as described above, both aliquots were warmed in a water bath at 37°C for 30 minutes and protected from light with tinfoil, defatted, digested, stored at -80°C overnight, and analyzed via microbial assay the next day.

- Storage for 4 months at -80°C

One aliquot containing whole milk was frozen as is at -80°C in an opaque storage box, and one aliquot was frozen after adding 1% w/v sodium ascorbate and mixing for 15 seconds using a vortex. After 4 months, as described above, both aliquots were warmed in a water bath at 37°C for 30 minutes and protected from light with tinfoil, defatted, digested, stored at -80°C overnight, and analyzed via microbial assay the next day.

The difference in folate concentration was compared between the baseline sample and samples stored for 4-months at -20°C and -80°C, with and without added sodium ascorbate. Because samples were paired, a paired t-test was used. All analyses were completed in Stata Version 15.

For samples stored at -80°C, there was no difference in the percent difference folate concentration between fresh milk values and samples at 4-months, independent of whether sodium ascorbate was added to the samples (see below). Similarly, there was no difference in percent difference between samples stored with and without sodium ascorbate at -20°C. However, at -20°C, we did observe that the folate concentration increased at 4-months relative to baseline. This is likely due to the presence of bacteria capable of producing folate in the milk, which were not killed at -20°C. Overall, we conclude that storing human milk at -80°C without sodium ascorbate does not affect folate concentration.

Percent difference between human milk values for samples analyzed fresh, and after storage with and without sodium ascorbate for 4-months in a -20 or -80 °C freezer

| **Comparison** | **Difference (%)** | ***P*-value** |
| --- | --- | --- |
| -20°C freezer |  |  |
| Baseline versus 4-months stored with sodium ascorbate | 20.0 | 0.05 |
| Baseline versus 4-months stored without sodium ascorbate | 12.4 | 0.05 |
| 4-months stored with sodium ascorbate versus without | 6.5 | 0.86 |
| -80°C freezer |  |  |
| Baseline versus 4-months stored with sodium ascorbate | 4.3 | 0.14 |
| Baseline versus 4-months stored without sodium ascorbate | 2.8 | 0.76 |
| 4-months stored with sodium ascorbate versus without | 6.3 | 0.96 |

*Human milk micronutrient inter-assay variation*

**Supplemental Table 4.** Precision and accuracy of human milk micronutrient analysis^1^

|  | **Precision (pooled samples)** | | **Accuracy** | | | **Certified value** | |
| --- | --- | --- | --- | --- | --- | --- | --- |
| **Micronutrient** | **Mean ± SD** | **%CV** | **SRM** | **Mean ± SD** | **%CV** | **Mean ± SD** | **Recovery (%)** |
| Iodine | 533 ± 30.3 µg/L | 5.7 | 1869a | 1.32 ± 0.12 mg/kg | 9.2 | 1.29 ± 0.06 mg/kg | 102 |
| Retinol | 0.445 ± 0.058 μg/mL | 4.9 | 1950 | 0.439 ± 0.044 μg/mL | 10.3 | 0.404 ± 0.035 μg/mL | 106 |
| α-tocopherol | 3.63 ± 0.25 μg/mL | 8.4 | 1950 | 8.25 ± 0.51 μg/mL | 6.2 | 8.18 ± 0.22 μg/mL | 101 |
| Vitamin B12 | 156 ± 11 pmol/L | 7.0 | 1869a | 292 ± 19 pmol/L | 4.3 | 330 ± 36 pmol/L | 88 |
| Folate | 102 ± 15 nmol/L | 15.0 | 1950 | 35.2 ± 4.3 nmol/L | 12.2 | 30.7 ± 0.1 nmol/L | 119 |
|  |  |  | BCR487 | 201 ± 28 ng/mL | 13.9 | 187 ng/mL | 107 |

^1^CV, coefficient of variation; SRM, standard reference material

*Maternal blood compositional analysis*

**Supplemental Table 5.** Characteristics of assays used for maternal serum compositional analysis^1^

| **Assay** | **Kit used** | **Inter-assay CV (%)** | **Duplicate measurements**  **n(%)** | **Standard reference material** | **Quality control methods** |
| --- | --- | --- | --- | --- | --- |
| Vitamin A (retinol) | N/A (manual method) | 1.2% | 138/11861  (1.2%) | Sigma Retinol (95144) | Third party ClinChek® Serum Control, lyophil, for Vitamins, Level I, II, III used  (2 levels/batch) |
| Vitamin B12 | Roche Elecsys Vitamin B12 II (Reference # 07212771190) | N/A^2^ | 10/164  (6.1%) | Vitamin B12 assay calibrators (ELECSYS B12 CALSET II, Category # 7212780190) | Roche vitamin B12 controls (ELECSYS PRECICONTROL Varia (Bone & Anemia)  Category # 5618860190) |

^1^CV, coefficient of variation; N/A, not applicable

^2^All samples analyzed on one day

*Statistical transformations*

**Supplemental Table 6.** Distribution and/or method of transformation to study measures

| **Micronutrient** | **Normally distributed** | **Method of transformation** |
| --- | --- | --- |
| Human milk |  |  |
| Iodine | No | Log |
| Retinol | No | Square-root |
| α-tocopherol | No | Log |
| Vitamin B12 | No | Square-root |
| Folate | No | Log |
| Infant anthropometry |  |  |
| Length-for-age z-score | Yes | - |
| Weight-for-age z-score | Yes | - |
| Weight-for-length z-score | Yes | - |
| Middle-upper arm circumference-for-age z-score | Yes | - |
| Head circumference-for-age z-score | Yes | - |
| Maternal serum |  |  |
| Retinol | No | Log |
| Vitamin B12 | No | Log |

*Milk micronutrient analysis*

**Supplemental Table 7.** Summary of combinations in human milk micronutrients below IOM mother’s milk adequacy estimates (MAEs) for all study participants, and by study group^1^

| **Micronutrient/s** | **Both groups**  (n=186) | **MMS only**  (n=97) | **SoC**  **only**  (n=89) | ***P*-value** |
| --- | --- | --- | --- | --- |
|  | n (%) | | |  |
| Folate and α-tocopherol | 1 (0.5) | 0 | 1 (1) | 0.18 |
| Folate and vitamin B12 | 7 (4) | 6 (6) | 1 (1) |  |
| Folate, iodine, and retinol | 1 (0.5) | 0 | 1 (1) |  |
| Folate, iodine, and α-tocopherol | 2 (1) | 1 (1) | 1 (1) |  |
| Folate, iodine, and vitamin B12 | 14 (8) | 7 (7) | 7 (8) |  |
| Folate, retinol, and α-tocopherol | 3 (2) | 2 (2) | 1 (1) |  |
| Folate, retinol, and vitamin B12 | 4 (2) | 1 (1) | 3 (3) |  |
| Folate, α-tocopherol, and B12 | 11 (6) | 8 (8) | 3 (3) |  |
| Folate, iodine, retinol, and α-tocopherol | 3 (2) | 1 (1) | 2 (2) |  |
| Folate, iodine, retinol, and vitamin B12 | 15 (8) | 7 (7) | 8 (9) |  |
| Folate, iodine, α-tocopherol, and vitamin B12 | 9 (5) | 5 (5) | 4 (4) |  |
| Folate, retinol. α-tocopherol, and vitamin B12 | 31 (17) | 22 (23) | 9 (10) |  |
| Folate, iodine, retinol α-tocopherol, and vitamin B12 (all) | 85 (46) | 37 (38) | 48 (54) |  |

^1^IOM, Institute of Medicine; MMS, multiple micronutrient supplement; SoC, standard of care

**Supplemental Table 8**. Complete table of associations between infant anthropometric measures and individual milk micronutrient concentrations or the number of milk micronutrient concentrations below the IOM MAEs^1^

| **Characteristics** | **N** | **β** | | **Unadjusted**  ***P*-value** | **β** | **Adjusted**^2^  ***P*-value** |
| --- | --- | --- | --- | --- | --- | --- |
| Length-for-age z-score (LAZ) |  |  | |  |  |  |
| Individual milk micronutrient concentrations |  |  | |  |  |  |
| Iodine | - | -0.0007 | | 0.36 | -0.0006 | 0.44 |
| Retinol | - | 0.52 | | 0.21 | 0.48 | 0.26 |
| α-tocopherol | - | 0.10 | | 0.03 | 0.10 | 0.03 |
| Vitamin B12 | - | 0.0001 | | 0.89 | 0.00001 | 0.95 |
| Folate | - | 0.005 | | 0.18 | 0.005 | 0.14 |
| Individual milk micronutrient IOM MAEs^3^ |  |  | |  |  |  |
| Iodine <141 μg/L | 129 | -0.005 | | 0.98 | -0.04 | 0.84 |
| Retinol <0.485 μg/mL | 142 | -0.40 | | 0.06 | -0.39 | 0.08 |
| α-tocopherol <4.9 μg/mL | 145 | -0.62 | | 0.003 | -0.62 | 0.003 |
| Below MAEs for all five milk micronutrients (IOM) | 85 | -0.37 | | 0.03 | -0.39 | 0.03 |
| Number of concurrent micronutrient deficiencies^34^ | *-* | *-* | | 0.20 | - | 0.45 |
| 5 | 85 | (ref) | | - | (ref) | - |
| 4 | 58 | 0.23 | | 0.22 | 0.26 | 0.17 |
| 2-3 | 43 | 0.56 | | 0.02 | 0.56 | 0.02 |
| Weight-for-age z-score (WAZ) |  |  | |  |  |  |
| Individual milk micronutrient concentrations |  |  | |  |  |  |
| Iodine | - | -0.001 | | 0.27 | -0.001 | 0.42 |
| Retinol | - | 0.33 | | 0.38 | 0.26 | 0.49 |
| α-tocopherol | - | 0.03 | | 0.61 | 0.03 | 0.61 |
| Vitamin B12 | - | -0.0004 | | 0.63 | -0.0006 | 0.54 |
| Folate | - | -0.004 | | 0.13 | -0.003 | 0.29 |
| Individual milk micronutrient IOM MAEs^3^ |  |  | |  |  |  |
| Iodine <141 μg/L | 129 | 0.04 | | 0.85 | -0.02 | 0.90 |
| Retinol <0.485 μg/mL | 142 | -0.25 | | 0.24 | -0.23 | 0.27 |
| α-tocopherol <4.9 μg/mL | 145 | -0.20 | | 0.33 | -0.18 | 0.37 |
| Below MAEs for all five milk micronutrients (IOM) | 85 | -0.22 | | 0.22 | -0.23 | 0.18 |
| Number of concurrent micronutrient deficiencies^4^ |  |  | |  |  |  |
| 5 | 85 | (ref) | | - | (ref) | - |
| 4 | 58 | 0.22 | | 0.26 | 0.23 | 0.22 |
| 2-3 | 43 | 0.21 | | 0.36 | 0.23 | 0.33 |
| Weight-for-length z-score (WLZ) |  |  | |  |  |  |
| Individual milk micronutrient concentrations |  |  | |  |  |  |
| Iodine | - | -0.0005 | | 0.54 | -0.0003 | 0.73 |
| Retinol | - | -0.10 | | 0.82 | -0.16 | 0.71 |
| α-tocopherol | - | -0.08 | | 0.20 | -0.08 | 0.18 |
| Vitamin B12 | - | -0.0007 | | 0.50 | -0.0008 | 0.42 |
| Folate | - | -0.01 | | 0.0001 | -0.01 | 0.002 |
| Individual milk micronutrient IOM MAEs^3^ |  |  | |  |  |  |
| Iodine <141 μg/L | 129 | 0.04 | | 0.84 | -0.01 | 0.96 |
| Retinol <0.485 μg/mL | 142 | 0.10 | | 0.61 | 0.11 | 0.55 |
| α-tocopherol <4.9 μg/mL | 145 | 0.44 | | 0.02 | 0.47 | 0.03 |
| Below MAEs for all five milk micronutrients (IOM) | 85 | 0.11 | | 0.54 | 0.11 | 0.54 |
| Number of concurrent micronutrient deficiencies^4^ |  |  | |  |  |  |
| 5 | 85 | (ref) | | - | (ref) | - |
| 4 | 58 | 0.05 | | 0.81 | 0.05 | 0.82 |
| 2-3 | 43 | -0.33 | | 0.15 | -0.32 | 0.16 |
| Head circumference-for-age z-score (HAZ) |  |  | |  |  |  |
| Individual milk micronutrient concentrations |  |  | |  |  |  |
| Iodine | - | -0.001 | | 0.11 | -0.001 | 0.23 |
| Retinol | - | 0.60 | | 0.17 | 0.54 | 0.23 |
| α-tocopherol | - | 0.09 | | 0.11 | 0.09 | 0.10 |
| Vitamin B12 | - | 0.0002 | | 0.76 | 0.0001 | 0.90 |
| Folate | - | -0.0009 | | 0.79 | -0.0001 | 0.97 |
| Individual milk micronutrient IOM MAEs^3^ |  |  | |  |  |  |
| Iodine <141 μg/L | 129 | 0.15 | | 0.38 | 0.08 | 0.65 |
| Retinol <0.485 μg/mL | 142 | -0.33 | | 0.11 | -0.30 | 0.14 |
| α-tocopherol <4.9 μg/mL | 145 | -0.33 | | 0.08 | -0.32 | 0.09 |
| Below MAEs for all five milk micronutrients (IOM) | 85 | -0.29 | | 0.08 | -0.32 | 0.05 |
| Number of concurrent micronutrient deficiencies^4^ |  |  | |  |  |  |
| 5 | 85 | (ref) | | - | (ref) | - |
| 4 | 58 | 0.33 | | 0.07 | 0.37 | 0.04 |
| 2-3 | 43 | 0.24 | | 0.27 | 0.25 | 0.25 |
| Middle-upper arm circumference-for-age z-score (MUAZ) | |  |  |  |  |  |
| Individual milk micronutrient concentrations |  |  | |  |  |  |
| Iodine | - | -0.001 | | 0.05 | -0.001 | 0.19 |
| Retinol | - | -0.24 | | 0.61 | -0.27 | 0.57 |
| α-tocopherol | - | -0.04 | | 0.48 | -0.04 | 0.51 |
| Vitamin B12 | - | -0.0001 | | 0.85 | -0.0003 | 0.67 |
| Folate | - | -0.002 | | 0.53 | -0.0007 | 0.81 |
| Individual milk micronutrient MAEs^3^ |  |  | |  |  |  |
| Iodine <141 μg/L | 129 | 0.24 | | 0.28 | 0.15 | 0.53 |
| Retinol <0.485 μg/mL | 142 | 0.05 | | 0.85 | 0.02 | 0.93 |
| α-tocopherol <4.9 μg/mL | 145 | -0.005 | | 0.98 | 0.001 | 1.0 |
| Below MAEs for all five milk micronutrients (IOM) | 85 | -0.03 | | 0.87 | -0.07 | 0.71 |
| Number of concurrent micronutrient deficiencies^4^ |  |  | |  |  |  |
| 5 | 85 | (ref) | | - | (ref) | - |
| 4 | 58 | 0.12 | | 0.58 | 0.16 | 0.45 |
| 2-3 | 43 | -0.10 | | 0.72 | -0.05 | 0.85 |

^1^IOM, Institute of Medicine; MAE, mother’s milk adequacy estimate

^2^Adjusted for maternal BMI category and number of breastmilk feeds in a day

^3^Data not presented for vitamin B12 and folate because ≥95% of participants were below the MAEs

^4^All participants had milk with folate below the cut-off, therefore the minimum number of concurrent milk concentrations below the MAEs was 2. However, as this was only experienced by 8 participants, having 2 or 3 milk concentrations below the MAEs was combined (see Supplemental Table 7 for exact breakdown).
